#  Frequency of monoclonal B-cell lymphocytosis in relatives of patients with chronic lymphocytic leukemia 

**Published:** 2016-06-30

**Authors:** Rossana Villegas Gracia, Catalina Franco Alzate, Javier Rendón Henao, José Domingo Torres Hernández, Patricia Elena Jaramillo Arbelaez

**Affiliations:** 1 Facultad de Ciencias de la Salud. Universidad de Córdoba. Monteria, Colombia; 2 Facultad de Medicina Universidad CES. Medellin, Colombia; 3 Facultad de Medicina Universidad de Antioquia. Medellin, Colombia; 4 Escuela Microbiología. Universidad de Antioquia. Medellin, Colombia

**Keywords:** Monoclonal B-cell lymphocytosis, chronic lymphocytic leukemia, B cells, flow cytometry

## Abstract

**Introduction:**

Monoclonal B-cell lymphocytosis is a symptom free condition characterized by the circulation of small clonal population of B lymphocytes in peripheral blood (less than 5x10^9^/L) expressing an immunophenotype similar to chronic lymphocytic leukemia. Different studies based on big hospital series have manifested a higher risk in subjects with monoclonal B-cell lymphocytosis to progress to a chronic lymphocytic leukemia. The behavior of this hematologic entity is unknown therefore its frequency in sporadic chronic lymphocytic leukemia patient relatives was determined.

**Methods:**

Transversal descriptive study, 8 color flow cytometry was performed using two of the tubes of the Euro Flow recommended panel, with modifications, for the diagnose of chronic lymphoproliferative disorders of B lymphocytes; besides, a fluorescence in situ hybridization was performed. univariate and bivariate analyses of the information were performed.

**Results:**

Monoclonal B-cell lymphocytosis frequency found in 51 analyzed relatives was 2%, it was a female participant, 59 years old, with a total leukocyte count of 7.7x10^9^/L and a B lymphocyte count of 0.124x10^9^/L; from these, 0.04x10^9^/L were clonal cells with restrictions of the kappa light chain. Rearrangements of the IGH gene (14q32) were found.

**Conclusion:**

Monoclonal B-cell lymphocytosis was detected in one relative of a patient with sporadic chronic lymphocytic leukemia in a frequency similar to the one reported in general population.

## Abstract (es)

**Introducción:**

La linfocitosis monoclonal de células B es una condición asintomática que se caracteriza por la circulación de pequeñas poblaciones clonales de linfocitos B en sangre periférica (menos de 5x109/L) que expresan un inmunofenotipo similar al de la leucemia linfoide cónica. Diferentes estudios basados en grandes series hospitalarias, han puesto de manifiesto un riesgo más elevado de los sujetos con linfocitosis monoclonal de células B de progresar a una leucemia linfoide crónica. En Colombia se desconoce el comportamiento de esta entidad hematológica, por tal razón se determinó su frecuencia en familiares de pacientes con leucemia linfoide crónica esporádica.

**Métodos:**

Estudio descriptivo transversal, se realizó citometría de flujo de 8 colores utilizando dos de los tubos del panel recomendado por Euro Flow para el diagnóstico de enfermedades linfoproliferativas crónicas de linfocitos B con modificaciones, además se hizo hibridación fluorescente *in situ*. Se realizó análisis univariado y bivariado.

**Resultados:**

La frecuencia de linfocitosis monoclonal de células B encontrada en los 51 familiares analizados fue del 2%, se trató de un participante del sexo femenino y 59 años de edad, con un recuento total de leucocitos de 7,7x10^9^/L y un recuento de linfocitos B de 0,124x10^9^/L; de estos 0,04x10^9^/L eran células clonales con restricción de la cadena ligera kappa. Se encontraron reordenamientos del gen IGH (14q32).

**Conclusión:**

Se detectó linfocitosis monoclonal de células B en un familiar de paciente con leucemia linfoide cónica esporádica en una frecuencia similar a la informada en la población general.

## Introduction

B Lymphocytes are immune system cells and primary effectors of humoral immunity, they specialize on synthesizing and secreting immunoglobulins (Ig) and originate from pluripotent hematopoeitic stem cells 1. Chronic lymphocytic leukemia (CLL) was placed in the World Health Organization (WHO) 2 classification among mature B cell neoplasms and characterizes by the accumulation of mature B lineage lymphoid cells but immunologically incompetent in peripheral blood , bone marrow, lymph nodes, spleen and other tissues. Its diagnose, in absence of extramedullary tissue infiltration, requires a sustained monoclonal lymphocytosis higher to 5x10^9^/L in peripheral blood with a characteristic immunophenotype when evaluated with flow cytometry which consists of positive CD19, weak CD20, aberrant co-expression of CD5, CD23 and weak expression of surface immunoglobulyns 3-5.

 Chronic lymphocytic leukemia causes are unknown, nevertheless it is known that the existence of family history is one of the risk factors which dispose to suffer this disease 6. First-degree relatives of patients with pathology have 7.5 times more risk than general population to suffer this or other lymphoid neoplasias 7, and in other cases, and in general population, it is possible to demonstrate by flow cytometry the presence in peripheral blood of a clonal population of B lymphocytes, a condition known as monoclonal B-cell lymphocytosis (MBL) 8. 

 Monoclonal B-cell lymphocytosis is an entity characterized by the presence of clonal populations of B lymphocytes in peripheral blood in a proportion minor to 5x10^9^/L 3, in subjects that do not present clinical signs or symptoms of a B cell chronic lymphoproliferative disorder 9. According to current classification, there are two types of MBL, CLL and non-CLL type; being CLL type the most frequent (75 % of the cases) 9. In this last one, clonal B cells present immunophenotypic characteristics identical to the ones observed in CLL and previously described. It has been found that in MBL CLL type the number of circulating cloned B cells may vary allowing a sub classification in clinic MBL (cMBL) and low count MBL. The first is characterized by the presence of lymphocytosis and a clonal B cell concentration equal or higher to 1.5x10^9^/L; and the second is detected during screening studies using sensitive techniques, and it has a clonal B cell count minor to 0.05x 10^9^/L 9.

Several studies have reported variable MBL prevalence highly depending on the characteristics of the examined population and the detection methods used for its identification; it is estimated that approximately 10 to 15% of people with lymphocytosis have MBL 10. Prevalence in adults in general (without including those with family history of CLL) oscillates between 0.12 and 14.3% 11. Studies performed in healthy population using two channel fluorescence cytometry detected an MBL prevalence of 0.12% 12, studies using four fluorochromes informed a prevalence of 3.5 to 3.8% 13,14, and those researches with five and eight colors reported 7.7 % and 12.0% respectively 9,15. When studying people with familial CLL (condition characterized by the presence of two or more people with diagnosed CLL in the same family) prevalences are high independently the flow cytometry technique used, for example, Rawstron *et al.* 16, used four color flow cytometry and identified, MBL in 13.5% of relatives in the first degree of consanguinity with patients with this type of CLL. It was also possible to establish the increment in the MBL cases while the age of the studied people increases 13,15. Based on these reports it has been possible to identify four parameters as the most determinant of the prevalence of MBL: a) first, the presence or absence of absolute lymphocytosis; second, the age of the people subjected to study; third, the detection sensitivity of the flow cytometry technique used; and fourth, the presence or absence of CLL in the family history 17.

Several studies based on large groups of hospital patients of CLL 18,19 have shown a higher risk of MBL subjects to become CLL patients. Proof this is the prospective cohort research by Landgren *et al*. 19, which demonstrated the hypothesis that CLL is always preceded by MBL. In this one, B cell clones were detected in 44 out of 45 samples (98%) of cryopreserved lymphocytes obtained in blood of subjects without cancer to whom CLL was later diagnosed. This precursor state lasted from 6 months to 6 years before leukemia developed. No reports providing data on MBL epidemiology and dynamic were found in Colombia, therefore the behavior of this hematologic entity in the country, and in particular in Medellín, is unknown. Hematopoietic origin cancer (leukemias and myelomas) occupied the ninth position in morbidity between 2007 and 2009 in Antioquia, with a gender distribution slightly higher in men, to whom 53% of cases were attributed 20; besides, leukemias were among the first ten dead causes in the 18 to 24 year old group 21. Thus it is important to know the MBL frequency in relatives of sporadic CLL patients assisted in health institutions in Medellín, and to know their clinical and biological characteristics; this would permit a following of this group and to take preventive and control measures which would lead to an early diagnose of a lymphoproliferative disorder. The following work determined monoclonal B-cell lymphocytosis frequency in sporadic chronic lymphocytic leukemia patient relatives.

## Materials and Methods

### Study type

Transversal descriptive study.

### Sujects

A non-probabilistic sample of convenience was performed in 51 relatives in the first and second-degree of consanguinity of 15 patients diagnosed with CLL, These were obtained during the 9 months stipulated for sample collection. Each patient filled out a survey to obtain epidemiologic and clinical data which would allow to verify the accomplishment of the selection criteria. Inclusion criteria were: men and women older than 18 years, who were relatives in the first or second degree of consanguinity of patients diagnosed with sporadic CLL without considering the stage that were not diagnosed nor presented symptoms of infectious, immunologic diseases or any kind of cancer. The exclusion criteria were the people who did not accept nor signed the informed consent.

### B-cell lymphocytosis detection

The following diagnose criteria were used 22:

1) Detection of a monoclonal B-cell population in the peripheral blood with light chain restriction

2) Presence of a disease-specific immunophenotype

3) Absolute B-cell count less than 5 x 10^9^/L

4) No other features of lymphoproliferative disorder (no lymph nodes, spleen and/or liver enlargement, no B-symptoms, such

as fever, weight loss or nighttime sweating)

5) No autoimmune or infectious disease .

### Flow cytometry

Ten mililiter of EDTA (Ethylenediaminetetraacetic acid) anticoagulated peripheral blood were taken, automated Complete blood count and flow cytometry analysis using two of the tubes (1 and 2) of the panel recommended by Euro Flow for the diagnose of chronic B-cell lymphoproliferative disorders 23 with some modifications were performed: (i) CD20-V450, CD45-V500c,smIgλ-fluorescein isothiocyanate (FITC), smIgk-Phycoerythrin (PE), CD5- peridinin-chlorophyll-protein-R-Phycoerythrin cyanine 5.5(PERCPCY5.5), CD19-Phycoerythrin cyanine 7(PECY7), smCD3-Allophycocyanin (APC), CD38-Allophycocyanin H7(APCH7); (ii) CD20-V450, CD45-V500c,CD23-fluorescein isothiocyanate (FITC), CD10-Phycoerythrin (PE), CD19-Phycoerythrin cyanine 7(PECY7), CD200-Allophycocyanin (APC), CD43-Allophycocyanin H7(APCH7) (Becton Dickinson Biosciences, BDB). Sample was washed using 300 μL of total blood with 10 mL of buffer BSA (BDB), and after this process 50 μL of washed and suspended blood were taken and mixed with the volume corresponding to each monoclonal antibody according to the manufacturer. Data acquisition was performed on a FACSCanto II flow cytometer (BDB), with previous quality control with pearls CS&T (BDB) to control de cytometer functioning and to control standardization to verify readings were made inside the corresponding fluorescence range. Analysis was made in two consecutive steps. In the first step information corresponding to the total cellularity of the sample 1x10^5^ cells approximately was stored, while in the second step only the information corresponding to B lymphocytes selected through an enriched window of CD19+ and/or CD20+ cells was stored, until acquiring the maximum possible number of events. A positive value higher to 30% 24 was considered for the CD38 analysis. Data analysis was performed using computer software Infinicyt by Cytognos.

### Fluorescence in situ hybridization (FISH)

Cytogenetic analysis was performed in the sample compatible with B cells monoclonal lymphocytosis by flow cytometry; EDTA anticoagulated peripheral blood was sent to a laboratory of cytogenetic were FISH was done using the following probes: Vysis LSI ATM (11q22.3) Spectrum Orange Probe; Vysis LSI TP53 (17p13.1) Spectrum Orange Probe; Vysis LSI D13S25 (13q14.3) Spectrum Orange Probe; Vysis 13q34 Spectrum Green FISH Probe Kit; CEP 12 Spectrum Orange Direct Labeled Fluorescent DNA Probe Kit; Vysis LSI IGH Dual Color, Break Apart Rearrangement Probe (Abbott Molecular); all these intended to detect deletions in 11q22.3, 13q14.3 and 17p13.1, rearrangement on 14q32 and trisomy 12 25.

### Statistical analysis

To describe gender, age and complete blood count parameters (univariate analysis) summary and measures and frequencies were calculated; to compare total leukocyte and lymphocyte counts with variables such as gender, kinship, current disease, disease history and medicine consumption (bivariate analysis) Mann-Whitney's U test was used, with previous verification of non-accomplishment of normality presumption by means of ShapiroWilk's test. Data was stored and analyzed with software SPSS^®^ version 19.0, significant *p* values lower to 0.05 were considered in all analyses. 

### Ethical consideration

The project was approved by the Ethic Committee of the Faculty of Dentistry of the University of Antioquia. The process of inclusion and voluntary participation of subjects was done according to national parameters (Resolution N° 008430 October 4 1993, Republic of Colombia, Health Minister Office) considered of minimal risk, and international (Helsinki Declaration and amendments, World Medical Association (WMA), Edinburgh, Scotland, October 2008), by which informed consent of all participants and patients with CLL was obtained and right to autonomy and confidentiality was respected.

## Results

### Study group characteristics

Twenty participants (39.2%) were men and 31 (60.7%) women, with an average age of 47.3±10 and a range between 26 and 66 years; 30% of the studied people were brothers or sisters of the patient with CLL, while 70% were sons or daughters. Leukocyte average in the studied participants was 7.32 ±1.78x10^9^/L (range= 4.8-12.85x10^9^/L); total lymphocytes showed an average of 2.43x10^9^/L (range= 0.99-4.5x10^9^/L). When comparing total blood tests results, significant differences were found (*p*= 0.030) in the total number of leukocytes in men and women, with significant higher values in women, this same was observed in the count of lymphocytes and platelets; nevertheless, no significant differences were found in the hematological variables when compared by kinship, current disease, disease history and medicine consumption. When studying the CD38 expression percentage in B lymphocytes in the study group, a mean expression of 15% (range= 2.6%-35.3%).

###  Monoclonal B-cell lymphocytosis frequency

 Monoclonal B-cell lymphocytosis was detected in one (2%) out of the 51 relatives analyzed, it was a 59 year old female participant with a total leukocyte count of 7.7x10^9^/L and B lymphocyte count of 0.124x10^9^/L; out of these 0.04x10^9^/L were clonal cells with kappa light chain restriction. CD38 expression in the cells of this participant was 6%. It was classified as low-count CLL-like MBL.

### Cytogenetic alteration detection with FISH

When detecting cytogenetic alterations in participants with MBL, rearrangement on 14q32 was found in 43% of the analyzed cells but neither deletions in 11q22.3, 13q14.3 and 17p13.1, nor trisomy 12 were detected.

## Discussion

 Monoclonal B-cell lymphocytosis frequency found in this research was 2%, which is lower to what has been reported in literature in studies performed in healthy people with familial CLL history. for example, Marti *et al.* 26, reported MBL in 18 % while Lanasa *et al*. 27, analyzed 622 people and their data was 16.2%. These reports could explain the lower frequency in the current work, mainly related with the kind of sporadic CLL in the families in Medellin. General rates of MBL among relatives of patients with this kind of leukemia may be compared to general population, but relatives older than 60 years show a higher risk of MBL, similar to what was observed in non-affected individuals from families with familial CLL 28. This suggests that MBL in these families represents an inherited predisposition to CLL and that both entities share factors of genetic origin. Increment in the risk of these relatives indicates CLL phenotype cells represent a surrogate marker of the carrier status 18.

Another aspect to consider with the frequency found is the age of the study group, as most of the analyzed subjects were between 40 and 50 years old and only 10% were older than 60 years. It has been reported that MBL behaves similar to CLL in older people. Nieto *et al*. 15, demonstrated that clonal B cells progressively incremented with age, being in people older than 90 years was 75%. Some researchers consider MBL could represent a normal aspect of the immune system, especially of the immunosenescence process due to the high frequency of the entity in older people which is 100 times higher than CLL's one 9.

Due to the low frequency of the event, the current work could neither study prevalence of subgroups nor explore possible associations between variables and the hematologic entity, nevertheless it is important to mention the positive case for MLB was female gender. Different to CLL, in which male-to-female ratio is 2:1 29, studies in MBL have not been conclusive in that respect. Results of a meta-analysis of MBL prevalence 30 did not show significant differences in the risk to suffer MBL between men and women, and it was found that only in those works including people with other diseases there was a higher prevalence in women, which could not be attributed to gender but to the base disease. Unfortunately, included researches did not explore the possible confusing effect of gender, which would improve the internal validity of these and the quality of the conclusions related with this association.

Regarding the subtypes of MBL described in literature, low-count CLL-like MBL was detected; based in number and clinical criteria, this is a clearly different entity of clinic MBL, even though they share the characteristic phenotype. This intrinsic difference is also supported by the few studies on its biological characteristics when little populations of B cell like CLL in peripheral blood have been isolated and analyzed 31. According to what has been reported up to now in literature, in low count MBL there is overrepresentation of IGHV4-59/61 genes and increment in the presence of these genes has also happened in people with advanced age, which suggests it could simply reflect restriction of repertoire related with age in healthy individuals. This supports even more the chance that this subtype may simple represent one aspect of the physiologic process of immunosenescence. Repertory similar restrictions are well established for the peripheral pool of T cells, when diversity of T cell receptor (TCR) seems to collapse with age 32.

Chromosomal alteration detected in the relative with MBL was 14q32 rearrangement, which implies Ig heavy chain; This has been mainly described in multiple myeloma, nevertheless several studies have reported it with more and more frequency in CLL patients. The rearrangement consists in complex and heterogeneous translocations with the breakpoint involving either the switch of IGH or the V, D or J gene. The primary translocations are due to somatic hyper mutation or errors in the VDJ portion of the switch region recombination. The translocations include a promiscuous array of at least 20 nonrandom chromosomal partners and the characterization of these translocations has led to the identification of critical deregulated oncogenes (e.g. BCL2, cyclin D). In each translocation, a potent enhancer is juxtaposed to deregulated oncogenes. The most frequent IGH translocations include t(4; 14) (p16.3; q32.3), t(11; 14) (q13; q32.3) and t(14; 16) (q32.3; q23) 33. Due to the kind of probe used for the cytogenetic analysis (Vysis LSI IGH Dual Color, Break Apart Rearrangement Probe), which provides valuable information on the gene rearrangement, but does not specify the partner with which translocation occurred, analysis of this finding in our participant becomes harder. When reviewing literature, we did not find reports of this disorder in low count MBL, nevertheless it has been detected in multi clonal cases of clinic MBL and in monoclonal and multi clonal CLL cases 34. A similar behavior was reported in another work of Henriques *et al*. 35, in which this kind of low count MBL disorder was not detected, but was in high count MBL and CLL. This cytogenetic disorder has also been reported in negative CD5 MBL and marginal zone lymphoma 36. Kern *et al*. 37, did not find significant statistical differences in the presentation of this translocation when comparing MBL cohorts (do not specify subtype) and CLL. Although this translocation is considered unfavorable CLL prognosis 38, it is not possible, as it is in most cytogenetic disorders of CLL prognosis value, to interpolate the MBL results, specifically because of the few studies where an analysis of this is done and because it is still considered of low frequency. There is agreement in that gradual acquisition of genetic disorders may determine the progress rate, not only from high count MBL to CLL, but also form low count MBL to high count MBL and finally CLL 34. The concurrence of chronic anti genetic stimulation by specific B cell receptors (BCRs) may increase and accelerate the expansion of MBL clones, facilitate the acquisition of new genetic disorders and contribute to the progress of CLL 39.

When it comes to clinical management of individuals with MBL, this is different according to the characteristic subtype of the entity. In pragmatic terms, the general physician could follow up people with low count MBL, due to these individuals have a completely normal blood count and clinical experience have demonstrated in particular that progression of this group is very rare. Close surveying is not necessary for this kind of people and a yearly complete blood count test is enough and appropriated 40. The participant to whom MBL was detected was recommended yearly checkups with the hematologist co researcher of the current work and a total blood count to monitor total lymphocyte concentration in peripheral blood. 

It must be considered that the external validity of this study presents limitations due to it was not possible to calculate the size of the sample and perform probability sampling because, among other factors, of the lack of data in the country, and even Latin America, on the expected influence of the event, as well as the absence of an adequate sampling frame of the patients in Medellín. Despite these limitations, this work constitutes a first approximation to the frequency of this entity in Colombia and provides initial ideas and directions to further research which is necessary especially if population subgroups, such as CLL patient relatives, are studied.
